# Ergonomic Risks, Psychosocial Associations and Prevalence of Musculoskeletal Symptoms Among Laboratory Workers in a Malaysian Medical Research Institute

**DOI:** 10.3390/epidemiologia7050122

**Published:** 2026-09-01

**Authors:** Imanul Hassan Abdul Shukor, Mohd Faiz Ibrahim, Wei Fern Siew

**Affiliations:** 1 Institute for Health Management, National Institutes of Health, Ministry of Health Malaysia, Shah Alam 40170, Malaysia; 2 Environmental Health Research Centre, Institute for Medical Research, National Institutes of Health, Ministry of Health Malaysia, Shah Alam 40170, Malaysia; drfaizibrahim@moh.gov.my; 3 Public Health and Community Medicine Department, School of Medicine, IMU University, Kuala Lumpur 57000, Malaysia; weifern_siew@imu.edu.my

**Keywords:** work-related musculoskeletal disorders, ergonomic risk assessment, work engagement, lab technicians, Rapid Entire Body Assessment, Poisson GEE, UWES-17

## Abstract

Background/Objectives: Musculoskeletal disorders (MSDs) represent a significant occupational health concern among medical laboratory workers (MLWs) worldwide. However, in cross-sectional epidemiological assessments, these are more accurately characterized as self-reported musculoskeletal symptoms (MSSs). Despite growing awareness of physical ergonomic risk factors, the multifactorial etiology of these symptoms, such as psychosocial factors, remains incompletely understood in research laboratory settings. Consequently, this study aimed to determine the prevalence of MSSs among MLWs in a Malaysian research institute, assess physical ergonomic risk in their daily tasks, and evaluate the empirical association between MSSs and psychosocial work engagement. Methods: A cross-sectional study was conducted among MLWs at a Malaysian medical research institute. The 12-month prevalence of self-reported MSSs was assessed using the Nordic Musculoskeletal Questionnaire. Physical ergonomic risk was evaluated utilizing the Rapid Entire Body Assessment (REBA). Psychosocial work engagement was measured using the 17-item Utrecht Work Engagement Scale. Results: A total of 115 MLWs participated, with 73 (63.5%) reporting MSSs. The neck had the highest complaints (*n* = 43, 37.4%), followed by the lower back (*n* = 35, 30.4%) and shoulders (*n* = 33, 28.7%). Multivariable modified Poisson Generalized Estimating Equations revealed that physical ergonomic risk (REBA) was significantly associated with symptoms in the shoulder, upper back, and lower back. Female sex was independently correlated with the overall prevalence of MSSs (aPR = 1.39; 95% CI = 1.14–1.68), with marked associations observed for the neck and knees. Psychosocially, high “absorption” in work was significantly associated with upper back symptom prevalence (aPR = 1.57; 95% CI = 1.15–2.14). Conclusions: Ergonomic strain demonstrates a strong association with axial and shoulder symptoms among MLWs, while demographic characteristics and cumulative tenure correlate with specific extremity symptoms. Paradoxically, high psychological absorption emerges as a distinct correlate of upper back symptoms, likely due to prolonged postural neglect during deep concentration. Occupational health programs may benefit from combining targeted ergonomic accommodations with strategies designed to mitigate the potential physical vulnerabilities of highly absorbed personnel.

## 1. Introduction

Musculoskeletal disorders (MSDs) represent one of the most prevalent occupational health problems globally, affecting workers across diverse industries and occupational settings [1]. These disorders encompass a wide range of inflammatory and degenerative conditions affecting muscles, tendons, ligaments, joints, peripheral nerves, and supporting blood vessels, resulting in pain, discomfort, and functional impairment [2]. The burden of work-related MSDs extends beyond individual suffering to encompass substantial economic costs through reduced productivity, absenteeism, and healthcare expenditures [3]. In cross-sectional epidemiological assessments, these conditions are often more accurately characterized as self-reported musculoskeletal symptoms (MSSs), serving as a crucial early indicator of underlying ergonomic and occupational strain [4].

Medical laboratory workers (MLWs) constitute a specialized occupational group whose work involves unique physical and cognitive demands. Laboratory work typically requires prolonged periods of microscopy, pipetting, specimen handling, and computer-based data entry [5]. Crucially, these tasks are not only physically repetitive but also demand sustained psychological focus and meticulous attention to detail, often performed in constrained workspaces with limited postural variation [5]. Previous studies have documented significant MSS prevalence among laboratory workers globally, with rates varying from 58.9% to over 90% depending on the population studied and assessment methods employed [6,7,8].

The etiology of work-related MSSs is widely recognized as multifactorial, driven by complex interactions between physical, biomechanical and psychosocial occupational hazards [9,10]. Traditional ergonomic risk assessment tools such as the Rapid Entire Body Assessment (REBA) and Rapid Upper Limb Assessment (RULA) have been developed to systematically evaluate postural risk factors and guide intervention strategies [11,12]. REBA has gained widespread acceptance for its ability to assess whole-body postural risk across diverse occupational settings [13].

However, emerging evidence suggests that traditional ergonomic assessment tools may not fully capture the complexity of MSS risk, particularly in occupations characterized by static postures, repetitive micro-movements, and significant psychosocial demands [14,15]. Several studies have reported paradoxical findings where workers experience high MSS prevalence despite low to moderate physical ergonomic risk scores [16,17]. This discrepancy suggests that psychological mechanisms play a substantial role in physical symptom development [16].

One increasingly relevant psychosocial construct is work engagement, encompassing dimensions of vigor, dedication, and absorption. While traditionally viewed as a positive organizational metric, intense psychological work engagement may act as a double-edged sword in physically constrained environments [18]. Highly engaged or deeply “absorbed” workers who enter states of cognitive flow may unconsciously bypass ergonomic best practices, delay necessary micro-breaks, and suppress early somatic signals of muscle fatigue [19]. Consequently, this psychological over-commitment can inadvertently lead to prolonged static muscle loading and postural neglect, exacerbating physical strain [18,19].

In Malaysia, occupational health research has increasingly focused on MSSs across various sectors, yet comprehensive data exploring this biopsychosocial intersection among laboratory workers remain limited [20]. Understanding the nuanced risk factors for MSSs among Malaysian laboratory professionals is essential for developing targeted, modern prevention strategies that address both the workstation and the worker’s psychological state.

Therefore, this study aimed to

1. Determine the 12-month prevalence of MSSs among medical laboratory workers in a Malaysian research institute using the standardized Nordic Musculoskeletal Questionnaire (NMQ);
2. Assess physical ergonomic risk across daily laboratory tasks using the REBA method;
3. Empirically evaluate the independent associations of physical ergonomic risks and psychosocial work engagement dimensions with self-reported musculoskeletal symptoms.

## 2. Materials and Methods

### 2.1. Study Design and Setting

This cross-sectional study was conducted at a medical research institute in Malaysia. The institute comprises multiple specialized laboratories engaged in clinical research, diagnostic testing, and biomedical research activities. Seven distinct laboratory settings were selected for ergonomic assessment to represent the diversity of work environments and tasks performed by laboratory workers within the institute. The typical ergonomic risk characteristics identified in each laboratory unit during the initial ergonomic risk assessment are summarized in Table 1.

### 2.2. Study Population

The study population consisted of MLWs employed at the research institute who had been working in their current positions for at least one year. Inclusion criteria required participants to be actively engaged in laboratory work involving specimen handling, microscopy, pipetting, equipment operation, and data management. Participants were clustered within seven distinct laboratory units; personnel within each specific unit performed homogeneous laboratory procedures throughout their working hours.

**Figure 1:**
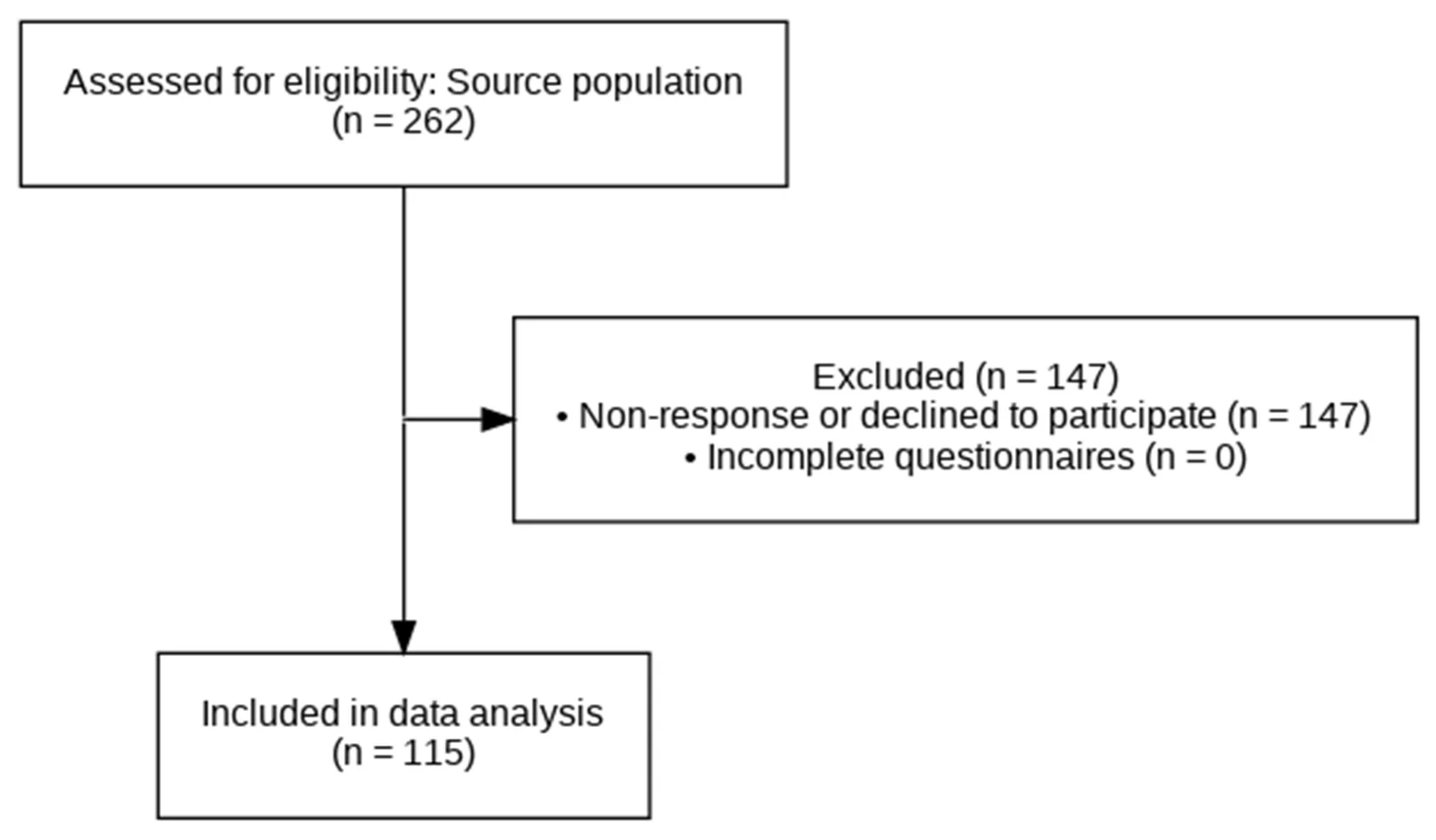
A flow diagram of the participant selection process.

A census sampling approach was utilized, inviting all eligible MLWs across the participating units. Out of a total eligible population of 262 workers, 147 individuals either declined to participate or did not respond. This resulted in a final analysis of 115 participants, yielding a response rate of 43.9%. Figure 1 shows the flow diagram detailing the initial source population, excluded individuals, and the final cohort included in the analysis.

### 2.3. Data Collection

Musculoskeletal symptoms (MSSs) were assessed using the English version of the Standardized Nordic Questionnaire (NMQ), a widely validated instrument for screening musculoskeletal symptoms across nine body regions: neck, shoulders, upper back, elbows, wrists/hands, lower back, hips/thighs, knees, and ankles/feet [21]. The NMQ has demonstrated excellent reliability and validity across diverse occupational populations and has been extensively used in studies of laboratory workers [6,8,21,22]. The questionnaire was self-administered by the participants via either paper-based or online format. The NMQ was utilized to determine the 12-month prevalence of musculoskeletal symptoms across nine distinct anatomical regions. Furthermore, the standard NMQ questions concerning activity limitations, specifically whether symptoms prevented the worker from performing normal work or daily activities, were included in the assessment. Data collection was carried out from November 2024 to April 2025, with all eligible MLWs in the research institute invited to participate in the study.

### 2.4. Ergonomic Risk Assessment

Ergonomic risk assessment was conducted using the Rapid Entire Body Assessment (REBA) method, a systematic observational tool designed to evaluate postural risk factors associated with work-related MSSs [11]. This validated and highly reliable REBA tool assesses body posture, force, repetition, and coupling to generate a risk score ranging from 1 to 15, with higher scores indicating greater ergonomic risk [11,13]. The classification for the risk level scoring is as follows:

- Score 1: Negligible risk, no action necessary;
- Scores 2–3: Low risk, change may be needed;
- Scores 4–7: Medium risk, further investigation and changes needed;
- Scores 8–10: High risk, investigate and implement changes soon;
- Scores 11–15: Very high risk, implement changes immediately.

Each MLW was assigned the REBA score corresponding to their primary laboratory unit. MLWs in this study were permanently assigned to a single unit and did not rotate between laboratories. Rather than assessing a single worker, observations were systematically conducted on three separate workers performing the three most frequent tasks, which were representative of that unit’s typical daily operations.

Data collection was scheduled during regular shifts, specifically between 9 a.m. and 3 p.m. Each of the three identified tasks was observed continuously for a duration of 10 to 15 min per worker. Video recordings and photographs were taken from multiple angles during this time to capture the full range of postural variations in the work cycle.

During the observation period, the most awkward, frequently repeated, or prolonged posture for each task was selected for detailed scoring. In addition to assessing the specific joint angles, the evaluation strictly incorporated the following REBA modifiers:

- Force/Load: Scored based on the weight of the objects handled or the physical exertion required (e.g., adding +1 for 5–10 kg, or +2 for >10 kg).
- Coupling: Evaluated based on the quality of the worker’s grip on the handled tools or containers (ranging from “good” to “unacceptable”).
- Activity: An activity score modifier (+1) was applied if one or more body parts were held in a sustained static posture for longer than one minute.

To reduce these multi-level observations into a single exposure metric for the laboratory unit, the following procedure was applied. First, a final REBA score was calculated for each of the three tasks performed by the three observed workers, resulting in nine individual REBA scores per laboratory. The final unit-level REBA score was then derived by calculating the median of these nine peak scores. This single aggregated score was subsequently assigned to all individuals working within that specific laboratory unit, serving as a standardized proxy for the unit’s shared ergonomic risk exposure.

To ensure measurement reliability and reduce subjective bias, the risk evaluation was conducted by three independent assessors. The primary observer holds formal credentials in advanced ergonomic risk assessment certified by the Department of Occupational Safety and Health (DOSH) Malaysia, and the two other assessors were trained and DOSH-certified in the REBA scoring protocol [23]. The assessors independently reviewed the visual media and assigned REBA scores to the identified high-risk postures.

To establish the final REBA score for each laboratory unit, a consensus rule was applied: a final score was recorded only when at least two of the three assessors assigned the exact same score. To evaluate the consistency of the ergonomic risk assessments among the three independent, DOSH-certified raters, an Intraclass Correlation Coefficient (ICC) was calculated. A two-way mixed-effects model based on absolute agreement and single measures was utilized. All statistical analyses regarding rater agreement were performed using SPSS version 29.0 [24].

### 2.5. Psychosocial Work Engagement

Psychosocial work engagement was assessed utilizing the 17-item Utrecht Work Engagement Scale (UWES-17) [25]. The UWES-17 measures work engagement as a positive, fulfilling, work-related state of mind characterized by three distinct subscales:

- Vigor (6 items; e.g., “At my work, I feel bursting with energy”);
- Dedication (5 items; e.g., “I am enthusiastic about my job”);
- Absorption (6 items; e.g., “Time flies when I am working”).

Participants responded to each item using a 7-point Likert scale ranging from 0 (“Never”) to 6 (“Always/Every day”). In accordance with the instrument’s scoring guidelines, mean scores were computed for each of the three subscales, with higher continuous scores indicating greater levels of vigor, dedication, and absorption. These continuous subscale scores were subsequently utilized as independent psychosocial predictors in the multivariable models.

### 2.6. Data Analysis

All statistical analyses were conducted using the Statistical Package for the Social Sciences (SPSS) for Windows, version 29.0 [24]. Descriptive statistics were used to summarize the demographic and occupational characteristics of the participants, as well as the prevalence of self-reported musculoskeletal symptoms. Continuous variables are presented as means and standard deviations (SDs), while categorical variables are reported as frequencies and percentages.

To evaluate the multifactorial associations between individual, physical, and psychosocial factors and the presence of musculoskeletal symptoms (MSSs), a multivariable Generalized Estimating Equations (GEE) model was utilized [26]. The GEE approach was specifically selected to account for the clustered nature of the study design, effectively adjusting for the non-independence of observations among workers who share the same laboratory environment and identical task-level REBA scores. An exchangeable working correlation matrix was specified to handle these within-unit dependencies. As the prevalence of MSSs is high according to previous studies, utilizing standard logistic regression risks artificially inflating the reported effect sizes. Therefore, a modified Poisson GEE approach was implemented by specifying a Poisson distribution with a log link function and utilizing a robust (empirical) covariance matrix estimator [27]. This mathematically rigorous method allows for the direct computation of adjusted Prevalence Ratios (aPRs) rather than Odds Ratios.

To evaluate the independent associations between physical ergonomic risk (REBA), dimensions of psychosocial work engagement (UWES-17), and musculoskeletal symptoms, we structured our analytical approach hierarchically. The primary, pre-specified confirmatory analysis consisted of a single multivariable modified Poisson GEE model evaluating the presence of overall musculoskeletal symptoms (MSSs) as the primary outcome. Following this, we conducted separate multivariable GEE models to investigate symptom prevalence across specific anatomical regions. These region-specific models were explicitly designated as secondary, exploratory analyses.

The primary outcome (MSS) was modeled as a binary response (0 = No symptoms, 1 = Has symptoms). The multivariable model simultaneously adjusted for demographic and physical covariates (gender, age, BMI, height, and years of work experience), aggregate physical ergonomic risk (REBA scores), and individual psychosocial factors derived from the UWES-17 (mean scores for vigor, dedication, and absorption) as main effects. All effect estimates are reported as aPRs with corresponding 95% confidence intervals (CIs). A two-tailed *p*-value of <0.05 was considered statistically significant.

## 3. Results

A total of 115 MLWs completed the survey (43.9% response rate), the majority of whom were female (80.9%, *n* = 93) and of Malay ethnicity (64.3%, *n* = 74). The Shapiro–Wilk test indicated that all continuous variables were non-normally distributed (*p* < 0.05). Accordingly, the median age of participants was 34 years (IQR = 30–39 years). Detailed sociodemographic characteristics are presented in Table 2.

### 3.1. Prevalence of Musculoskeletal Symptoms

The 12-month prevalence of MSSs among MLWs in the Malaysian research institute was substantial, with 63.5% (*n* = 73) of participants reporting musculoskeletal symptoms in at least one body region during the previous year. The distribution of MSSs across body regions revealed distinct patterns of involvement, with the upper body regions being predominantly affected.

The neck was the most affected body region, with a prevalence of 37.4% (*n* = 43). This was followed by the lower back at 30.4% (*n* = 35) and shoulders at 28.7% (*n* = 33). These findings indicate that the cervical, lumbar, and shoulder regions bear the greatest burden of MSSs among this occupational group. Table 3 shows the breakdown of MSSs from each body region, with the category of “any region” defined as the self-reported presence of musculoskeletal symptoms in at least one of the nine anatomical areas assessed.

Regarding activity limitations, 24.7% (*n* = 18) of the symptomatic workers reported that their musculoskeletal symptoms had prevented them from performing their normal work activities during the preceding 12 months.

### 3.2. Ergonomic Risk Levels

**Figure 2:**
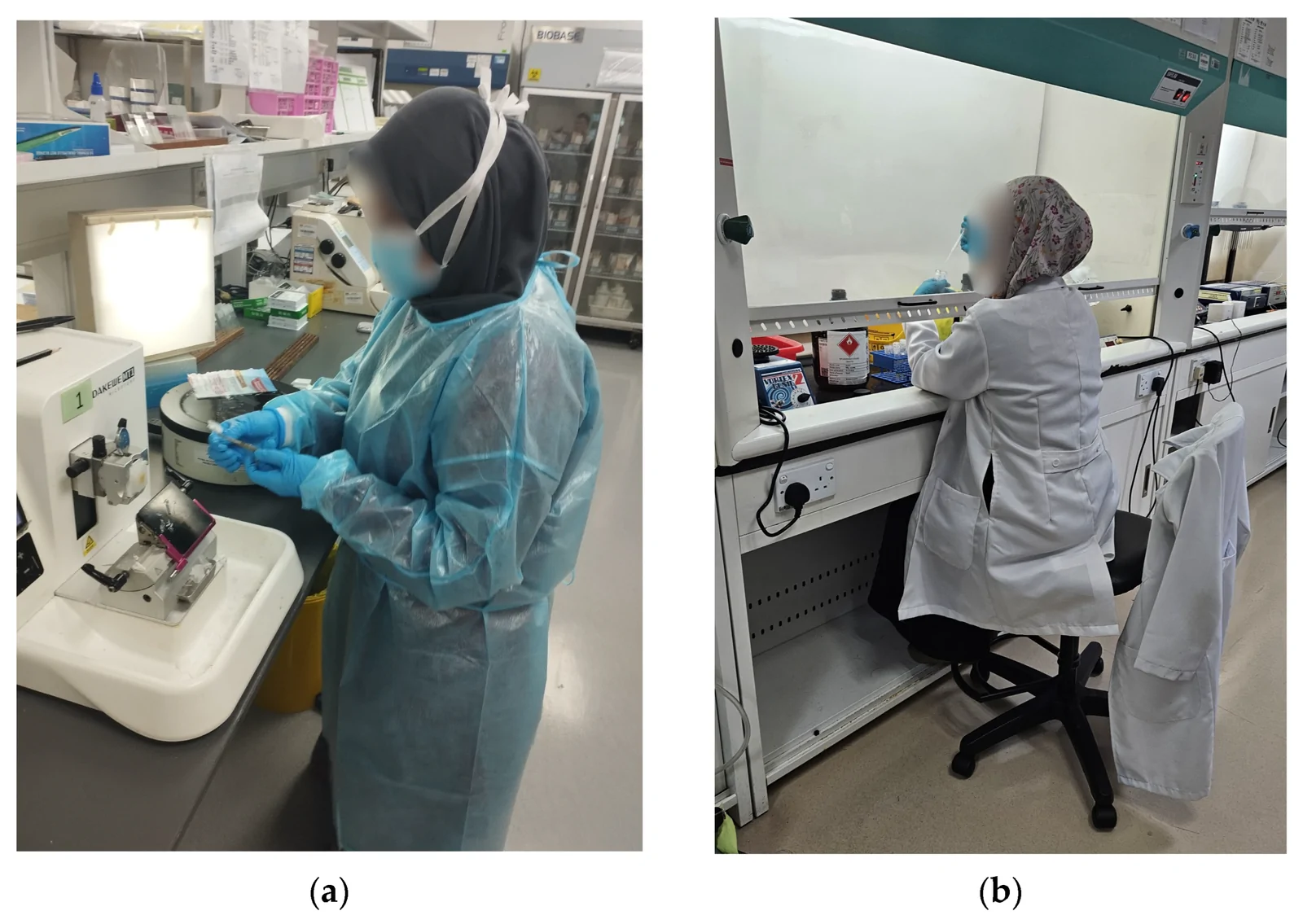
Examples of laboratory postures assessed using REBA. (**a**) An MLW performing a tissue extraction task in the Unit E laboratory. (**b**) An MLW conducting a pipetting task in the Unit G laboratory.

Ergonomic risk assessment using the REBA method was conducted across seven distinct laboratory settings within the research institute. The assessment evaluated typical work postures and activities performed by laboratory workers, including microscopy, pipetting, specimen handling, equipment operation, and computer-based documentation. Examples of postures evaluated using REBA during common laboratory activities are shown in Figure 2.

The REBA scores obtained across all seven laboratory settings were remarkably consistent, yielding scores of 3 and 4. The inter-rater reliability for the REBA scores was found to be good, with an ICC of 0.833 (95% CI: 0.543–0.965, *p* < 0.001). This indicates a high degree of agreement and minimal subjective bias across the assessors’ evaluations. Table 4 shows the REBA scores according to the laboratory units.

### 3.3. Work Engagement

Preliminary analyses were conducted to evaluate the reliability of the work engagement measures. The internal consistency for the subscales in this study was acceptable: α = 0.71 for vigor, α = 0.73 for dedication, and α = 0.72 for absorption. Overall, participants reported moderate-to-high levels of work engagement (M = 4.27, SD = 0.97). When examining the specific dimensions, participants scored highest on dedication (M = 4.68, SD = 1.05), followed by vigor (M = 4.21, SD = 1.06) and absorption (M = 3.99, SD = 1.14).

### 3.4. Multivariable Predictors of Musculoskeletal Symptoms

Regarding our primary pre-specified confirmatory analysis, the multivariable GEE model for overall musculoskeletal symptoms (MSSs) demonstrated that female sex was significantly associated with MSSs (aPR = 1.39; 95% CI = 1.14–1.68).

Following this primary analysis, we also conducted secondary exploratory multivariable models to examine specific anatomical regions. It is important to note that due to the number of statistical tests performed across different body regions, these isolated regional findings are treated as hypothesis-generating and should be interpreted with caution. In these exploratory regional analyses, we found that physical ergonomic risk, quantified by the REBA score, demonstrated the most consistent associations with symptoms, particularly concerning the axial skeleton and shoulders. A one-point increase in the REBA score was significantly associated with higher adjusted prevalence of symptoms in the shoulders (aPR = 1.39; 95% CI = 1.15–1.68), upper back (aPR = 1.79; 95% CI = 1.50–2.12), and lower back (aPR = 1.85; 95% CI = 1.04–3.30). Female sex was also a prominent correlate, demonstrating significant region-specific associations in the neck (aPR = 1.82; 95% CI = 1.05–3.16) and knees (aPR = 3.02; 95% CI = 1.13–8.02).

Additionally, anthropometric and occupational tenure variables exhibited region-specific biomechanical patterns. Greater body height demonstrated a slight inverse association with neck (aPR = 0.97; 95% CI = 0.95–0.99) and wrist/hand symptoms (aPR = 0.95; 95% CI = 0.90–0.99) but was significantly associated with a higher prevalence of knee symptoms (aPR = 1.04; 95% CI = 1.01–1.08). Furthermore, cumulative occupational exposure, measured by work experience, was exclusively associated with increased knee symptoms (aPR = 1.15; 95% CI = 1.03–1.29).

Psychosocial work engagement did not function as a uniform protective resource. The models revealed that the absorption subscale of the UWES-17 was independently associated with upper back symptoms, corresponding to a 57% higher adjusted prevalence per unit increase (aPR = 1.57; 95% CI = 1.15–2.14). The vigor and dedication subscales did not demonstrate statistically significant independent associations with MSS prevalence across the converging models. Due to sparse symptom prevalence and subsequent complete data separation resulting in singular Hessian matrices, models for the elbow, hip/thigh, and ankle/foot regions did not reach mathematical convergence and were consequently omitted from the multivariable analysis. Adjusted prevalence ratios (aPRs) and 95% confidence intervals (CI) for the overall occurrence of MSSs and specific body regions are presented in Table 5.

### 3.5. Multicollinearity Assessment

An examination of the parameter estimate covariances across the regional models reveals notable multicollinearity specifically between age and occupational tenure. Across all evaluated models, including overall MSSs, neck, shoulder, upper back, wrists/hands, lower back, and knee, the covariance between age and years of work experience is consistently negative and proportionally large relative to their individual parameter variances. For example, this covariance manifests as −0.00165 in any region MSS model, −0.00605 for the neck, and −0.01007 for the shoulder. This indicates that age and tenure share substantial overlapping variance.

Conversely, other evaluated variables demonstrate negligible collinearity. Notably, the covariance between height and BMI is virtually zero across all body regions. This confirms that height and BMI function as mathematically independent covariates within these GEE models without structural redundancy.

## 4. Discussion

This study utilized a robust, population-average Generalized Estimating Equations (GEE) framework to evaluate the biopsychosocial factors associated with musculoskeletal symptoms (MSSs) among medical laboratory workers (MLWs) [27]. The overall narrative arc of the findings challenges the assumption that psychosocial engagement is uniformly inversely associated with occupational strain. Instead, physical ergonomic exposure (REBA) emerged as the most consistent correlate of symptoms across the axial skeleton. Furthermore, demographic characteristics (gender), anthropometric mismatches (height), and cumulative temporal exposures (tenure) demonstrated highly specific regional biomechanical patterns. Most notably, psychosocial work engagement exhibited a complex relationship with MSSs, with deep cognitive absorption acting as a paradoxical, double-edged correlate of upper back symptoms.

### 4.1. MSS Prevalence in Context

The overall 12-month MSS prevalence of 63.5% observed in this study is substantial and aligns with prevalence rates reported in the international literature on laboratory workers [6,8,22]. The predominance of neck symptoms (37.4%) in our study is consistent with the well-documented pattern of cervical region involvement among laboratory workers, particularly those engaged in microscopy and computer-based tasks [7,28,29]. The high prevalence of neck symptoms likely reflects the sustained forward head posture and cervical flexion required during microscopy, specimen examination, and computer work [8,28,30,31].

Lower back pain, affecting 30.4% of participants in our study, represents another major area of concern. This prevalence is comparable to rates reported in studies from Ethiopia (33.6%) and Indonesia (47.9%) [8,29]. Lower back symptoms among laboratory workers have been attributed to prolonged standing, forward bending during specimen handling, and static loading of the lumbar spine during extended work periods [6,22,30,32].

The prevalence of shoulder symptoms observed in this study (28.7%) is identical to that reported in a similar cohort of Estonian laboratory workers [30]. These shoulder symptoms reflect the repetitive upper extremity movements and sustained shoulder positioning required for pipetting, specimen manipulation, and equipment operation [6,29]. The shoulder region is particularly vulnerable to overuse injuries in laboratory work due to the combination of repetitive movements, static muscle loading, and constrained workspace design [33].

### 4.2. Ergonomic Exposure as the Primary Driver of Axial Strain

The most consistent variable associated with MSSs in the axial skeleton and proximal upper extremities was the physical ergonomic risk, quantified by the REBA score. Specifically, higher REBA scores were significantly associated with an increased prevalence of symptoms in the shoulder (aPR = 1.39; 95% CI = 1.15–1.68), upper back (aPR = 1.79; 95% CI = 1.50–2.12), and lower back (aPR = 1.85; 95% CI = 1.04–3.30). These three regions are fundamentally tied to the biomechanical realities of standard laboratory tasks, which heavily involve static forward-leaning, repetitive lifting, and sustained reaching during microscopy or pipetting within biosafety cabinets [7,28,30]. This is a strong and coherent finding indicating that objective physical exposure correlates with MSSs more consistently across body regions than psychological states do. From an occupational health perspective, this suggests that direct, physical ergonomic interventions, such as implementing fully adjustable workstations, should serve as the primary preventive lever, with psychological engagement treated as a secondary or moderating factor [28,34,35].

### 4.3. Demographic Vulnerabilities and Anthropometric Mismatch

Female sex emerged as a consistent correlate, demonstrating a significant association with a higher overall prevalence of MSSs (aPR = 1.39; 95% CI = 1.14–1.68) and demonstrating striking region-specific susceptibilities, particularly in the neck (aPR = 1.82; 95% CI = 1.05–3.16) and knees (aPR = 3.02; 95% CI = 1.13–8.02). This disparity is frequently observed in numerous previous studies among medical laboratory workers, with women being about twice as likely to report symptoms as men in a Spanish cohort (OR = 2.09) and an Ethiopian cohort (OR = 1.89) [8,36]. In the laboratory setting, this elevated prevalence is hypothesized to be related to an anthropometric mismatch, as standard laboratory equipment, biosafety hoods, and workbench heights are often designed around male dimensional averages [7,8]. This design bias may force female MLWs into greater postural extremes and awkward compensations to perform standard tasks. Furthermore, this gender disparity may be compounded by the “double burden” of domestic and caregiving responsibilities outside of work hours, which limits essential physical recovery time for the musculoskeletal system [8]. Moreover, women generally have less baseline muscle mass and absolute physical strength compared to men, making them more vulnerable to muscle fatigue in physically demanding tasks [7,8].

### 4.4. Region-Specific Effects of Height and Cumulative Tenure

Anthropometric and temporal factors in this study exhibited distinct, localized biomechanical patterns rather than generalized systemic risks. Interestingly, taller stature demonstrated a direction-reversal effect: it demonstrated a mild inverse association with neck (aPR = 0.97; 95% CI = 0.95–0.99) and wrists/hands (aPR = 0.95; 95% CI = 0.90–0.99), yet was significantly associated with a higher prevalence of knee symptoms (aPR = 1.04; 95% CI = 1.01–1.08). This dichotomy is biomechanically plausible. Taller MLWs are likely to experience less cervical flexion and wrist deviation when viewing screens or working at standard bench heights [7]. However, they suffer increased patellofemoral loading when forced to stoop, splay their legs, or stand for prolonged periods at those same fixed-height stations [29].

Similarly, occupational tenure was exclusively associated with knee symptoms (aPR = 1.15; 95% CI = 1.03–1.29). This aligns seamlessly with a localized cumulative loading mechanism related to prolonged standing and joint stress over a career [8,22,37].

### 4.5. The Paradox of Work Engagement: Absorption as a Double-Edged Sword

A particularly noteworthy nuance of this study is the non-uniform behavior of psychosocial work engagement. Rather than acting as a universal protective resource, the absorption subscale was independently associated with a higher prevalence of upper back symptoms (aPR = 1.57; 95% CI = 1.15–2.14). Meanwhile, Dedication demonstrated a potential inverse association with the lower back and knees. This complicates the simplified hypothesis that engaged workers uniformly experience fewer symptoms, suggesting that the UWES-17 subscales are not interchangeable proxies for occupational well-being. To the best of the authors’ knowledge, this is the first study to investigate the complex association between specific dimensions of work engagement and musculoskeletal symptoms among medical laboratory workers.

Viewed through the lens of the Job Demands–Resources (JD-R) framework, the risk associated with high absorption likely reflects a “presenteeism” mechanism [38,39]. Highly absorbed workers, who frequently enter a state of deep cognitive flow and immersion in their tasks, may inadvertently sustain awkward, static postures for prolonged periods. In this flow state, they are more likely to delay necessary micro-breaks and cognitively suppress early somatic signals of muscle fatigue. Consequently, while absorption benefits organizational productivity, it may pose a distinct physical vulnerability, potentially allowing static loading on the upper back to accumulate into symptomatic tissue strain [40].

### 4.6. Methodological Limitations

Finally, several methodological limitations must be addressed. The study achieved a response rate of 43.9% (115 out of 262 eligible workers). This introduces the potential for non-response bias, commonly known as volunteer bias in health research [41]. It is possible that workers experiencing active musculoskeletal symptoms were more motivated to complete the questionnaire than asymptomatic workers, which may have resulted in an overestimation of the true symptom prevalence within the units.

Furthermore, due to sparse regional symptom prevalence and the resulting complete data separation, the multivariable GEE models for the elbows, hips/thighs, and ankles/feet failed to converge. Consequently, adjusted estimates for these three specific regions could not be modeled and were omitted. While their raw prevalence is reported descriptively, future multi-center studies with larger, pooled cohorts are required to robustly evaluate multivariable associations for these specific extremities.

Additionally, the cross-sectional design inherently limits the ability to establish strict temporal causality, particularly regarding the complex interplay between psychological absorption and the presence of physical symptoms [42]. This issue is compounded by a specific temporal mismatch between the assessment of musculoskeletal symptoms and the measurement of physical ergonomic risk. The Nordic Musculoskeletal Questionnaire (NMQ) captures self-reported symptoms experienced over the preceding 12-month recall period, whereas the REBA observations represent a cross-sectional snapshot of current physical exposure. Consequently, the ergonomic risk observed during the study period may not fully reflect the variations in tasks, cumulative physical load, or fluctuating exposures the workers experienced over the entire year. Furthermore, it is possible that workers already experiencing musculoskeletal discomfort may have subconsciously altered their postures or work habits to alleviate pain prior to our observations, which could lead to an underestimation of their historical exposure. Future longitudinal studies are needed to better align the timelines of ergonomic exposure with symptom development.

## 5. Conclusions

This study reveals a significant burden of musculoskeletal disorders among medical laboratory workers in a Malaysian research institute, with an overall 12-month prevalence of 63.5% and particularly high rates of neck (37.4%), lower back (30.4%), and shoulder (28.7%) symptoms. This study demonstrates that musculoskeletal symptoms (MSSs) among medical laboratory workers are associated with a complex, multifactorial interplay of physical, demographic, and psychological elements. Objective physical ergonomic risk remains the most consistent correlate of symptoms in the axial skeleton and shoulders. Furthermore, this physical risk is accompanied by demographic vulnerabilities, most notably potential gender-based anthropometric mismatches with standard laboratory workstations, as well as localized cumulative associations between prolonged occupational tenure and lower extremity symptoms.

Crucially, this research challenges the conventional paradigm that psychosocial work engagement is inversely associated with occupational strain. Instead, deep cognitive absorption emerges as a distinct, paradoxical correlate of upper back symptoms. This leads to the plausible hypothesis that highly focused workers may inadvertently experience postural neglect and sustained static loading during periods of deep concentration.

Consequently, occupational health interventions in laboratory settings should consider evolving beyond generic ergonomic training. Institutions are encouraged to prioritize fully adjustable, anthropometrically inclusive workstations to mitigate gender- and height-based biomechanical disadvantages. Furthermore, ergonomic programs may benefit from incorporating psychosocial strategies, specifically training highly engaged and “absorbed” personnel to recognize early somatic signals of muscle fatigue and encouraging regular micro-breaks to disrupt hazardous static postures. Ultimately, protecting the musculoskeletal health of modern laboratory professionals calls for a holistic, biopsychosocial approach that addresses both the physical constraints of the workstation and the psychological state of the worker.

## Figures and Tables

**Table 1:** Main work tasks, common ergonomic risks, and MLW distribution.

Laboratory Unit	Main Work Tasks	Common Ergonomic Risks	Total MLWs, *n* (%)
Unit A	Cell culture; viral load quantification.	Prolonged standing; static posture.	35 (30.4)
Unit B	Biochemical assay; metabolomics.	Repetitive pipetting; prolonged standing.	8 (7.0)
Unit C	Toxicology analysis; water quality testing.	Manual handling; awkward posture.	15 (13.0)
Unit D	Allergen test; cytokine profiling.	Awkward posture; repetitive pipetting.	19 (16.5)
Unit E	Molecular pathology; RNA sequencing.	Repetitive movements; prolonged sitting.	13 (11.3)
Unit F	Phytochemical test; bioassay.	Repetitive filtration; awkward posture.	7 (6.1)
Unit G	Genomic sequencing; gene expression analysis.	Static posture, repetitive movements.	18 (15.7)

**Table 2:** Sociodemographic characteristics.

Characteristics	Total Participants (*N* = 115)
**Age (years),** Median (IQR)	34 (30–39)
**Height (cm),** Median (IQR)	158 (153–163)
**Body Mass Index,** Median (IQR)	25.97 (21.91–29.38)
**Work Experience (years),** Median (IQR)	10 (5–15)
**Gender**	
Female	93 (80.9)
Male	22 (19.1)
**Ethnicity**	
Malay	74 (64.3)
East Malaysian	28 (24.3)
Others	13 (11.3)

**Table 3:** Prevalence of MSSs across body regions (*N* = 115).

Body Region	Had Symptoms, *n* (%)	95% CI (%)
Neck	43 (37.4)	28.6–46.9
Upper back	24 (20.9)	13.8–29.6
Shoulders	33 (28.7)	20.6–38.0
Elbows	2 (1.7)	0.2–6.1
Wrists/hands	30 (26.1)	18.3–35.1
Lower back	35 (30.4)	22.2–39.7
Hips/thighs	15 (13.0)	7.5–20.7
Knees	21 (18.3)	11.7–26.6
Ankles/feet	21 (18.3)	11.7–26.6
Any region	73 (63.5)	54.0–72.3

95% confidence interval (95% CI) was calculated using the Clopper–Pearson method. “Any region” refers to the presence of symptoms in one or more of the surveyed body parts.

**Table 4:** REBA scores across laboratory units.

Laboratory Center	REBA Score	Risk Level
Unit A	3	Low
Unit B	3	Low
Unit C	3	Low
Unit D	3	Low
Unit E	3	Low
Unit F	3	Low
Unit G	4	Medium

**Table 5:** Adjusted prevalence ratios (aPRs) with 95% confidence intervals for musculoskeletal symptoms according to body region.

Predictor	Any Region	Neck	Shoulder	Upper Back	Wrists/Hands	Lower Back	Knee
Female	1.39 (1.14–1.68) *	1.82 (1.05–3.16) *	1.70 (0.55–5.22)	3.00 (0.72–12.57)	1.35 (0.46–3.96)	2.08 (0.95–4.56)	3.02 (1.13–8.02) *
Age	0.95 (0.88–1.02)	0.97 (0.83–1.12)	0.98 (0.80–1.20)	1.03 (0.88–1.21)	1.00 (0.83–1.19)	0.99 (0.88–1.10)	0.93 (0.84–1.04)
Work experience	1.05 (0.96–1.15)	1.02 (0.88–1.20)	1.02 (0.84–1.23)	0.96 (0.86–1.09)	1.02 (0.85–1.24)	1.01 (0.92–1.12)	1.15 (1.03–1.29) *
Height	1.00 (0.98–1.02)	0.97 (0.95–0.99) *	0.97 (0.93–1.01)	1.02 (0.95–1.09)	0.95 (0.90–0.99) *	1.01 (0.97–1.05)	1.04 (1.01–1.08) *
Body mass index	1.00 (0.98–1.02)	1.02 (0.97–1.07)	0.98 (0.97–0.99) *	0.96 (0.89–1.03)	1.00 (0.95–1.06)	1.02 (0.99–1.06)	1.04 (0.98–1.10)
REBA score	1.14 (0.86–1.51)	1.11 (0.70–1.75)	1.39 (1.15–1.68) *	1.79 (1.50–2.12) *	1.22 (0.72–2.06)	1.85 (1.04–3.30) *	0.65 (0.41–1.01)
Vigor	0.97 (0.77–1.23)	1.02 (0.74–1.41)	0.96 (0.74–1.24)	1.02 (0.59–1.74)	0.70 (0.48–1.04)	0.93 (0.62–1.38)	0.80 (0.54–1.20)
Dedication	0.91 (0.75–1.09)	0.92 (0.64–1.33)	1.09 (0.80–1.47)	0.68 (0.44–1.06)	1.08 (0.78–1.48)	0.78 (0.48–1.28)	0.74 (0.36–1.51)
Absorption	1.11 (0.83–1.49)	0.93 (0.65–1.35)	1.13 (0.65–1.96)	1.57 (1.15–2.14) *	1.25 (0.79–1.96)	1.24 (0.74–2.08)	1.48 (0.86–2.55)

* A *p*-value of <0.05 is statistically significant with a 95% confidence interval. Data are presented as adjusted Prevalence Ratios (95% Wald CI) derived from modified Poisson GEE models. Male is the reference category for gender. Multivariable estimates for the elbow, hip/thigh, and ankle/foot regions were omitted due to model non-convergence resulting from sparse symptom prevalence and complete data separation.

## Data Availability

The raw data supporting the conclusions of this article will be made available by the authors upon request.

## Abbreviations

The following abbreviations are used in this manuscript:

MSSs	Musculoskeletal Symptoms
MLWs	Medical Laboratory Workers
NMQ	Nordic Musculoskeletal Questionnaire
REBA	Rapid Entire Body Assessment
BMI	Body Mass Index
SPSS	Statistical Package for the Social Sciences
GEE	Generalized Estimating Equations
aPR	Adjusted Prevalence Ratio
